# Preoperative Radiochemotherapy in Rectal Cancer: Is There an Impact of Oxaliplatin on Pathologic Complete Response and Survival Rates under “Real World“ Conditions?

**DOI:** 10.3390/cells12030399

**Published:** 2023-01-22

**Authors:** Alexander Grabenbauer, Thomas Aigner, Holger Göbel, Bernhard J. Leibl, Christof Lamberti, Gerhard G. Grabenbauer, Luitpold V. Distel

**Affiliations:** 1 Department of Radiation Oncology, Universitätsklinikum Erlangen, Friedrich-Alexander-Universität Erlangen-Nürnberg, 91054 Erlangen, Germany; 2 Department of Pathology, Coburg Cancer Center, 96450 Coburg, Germany; 3 Department of Gastroenterology, Coburg Cancer Center, 96450 Coburg, Germany; 4 Department of Abdominal Surgery, Coburg Cancer Center, 96450 Coburg, Germany; 5 Department of Hematology and Oncology, Coburg Cancer Center, 96450 Coburg, Germany; 6 Department of Radiation Oncology, Coburg Cancer Center, 96450 Coburg, Germany

**Keywords:** locally advanced rectal cancer, neoadjuvant radiochemotherapy, oxaliplatin, total neoadjuvant radiochemotherapy, survival, pathological complete remission

## Abstract

This study aimed to evaluate the benefit of additional administration of oxaliplatin during fluorouracil-based neoadjuvant radiochemotherapy (nRCT) in terms of pathologic complete remission (pCR), disease-free survival (DFS), and overall survival (OS) in patients with advanced rectal cancer. Between 2006 and 2021, 669 patients (pts) were diagnosed with locally advanced rectal cancer, of whom a total of 414 pts with nRCT were identified and included in the study. A total of 283 pts were treated by nRCT using concurrent chemotherapy with fluorouracil or capecitabine; 131 pts were treated using a combination of fluorouracil or capecitabine and oxaliplatin. Propensity score matching analyses (PSM) with 114 pts in each group were used to balance the patients’ characteristics. OS, DFS, pCR-rate, and potential prognostic factors were compared between the two groups. The median follow-up time was 59.5 weeks in the fluorouracil-group and 43 weeks in the fluorouracil/oxaliplatin group (*p* = 0.003). After PSM, the pCR-rate (including sustained clinical complete remission) was 27% (31/114 pts) in the fluorouracil/oxaliplatin group and 16% (18/114 pts) in the fluorouracil-group (*p* = 0.033). There was no difference between these two groups for both 10-year OS and DFS neither before nor after PSM, respectively (OS: 72.6% vs. 55.4%, *p* = 0.066, and 67.8% vs. 55.1%, *p* = 0.703, and DFS: 44.8% vs. 46.8%, *p* = 0.134, and 44.7% vs. 42.3%, *p* = 0.184). Multivariate analysis identified regression grading according to Dworak grade 4 (HR: 0.659; CI: 0.471–0.921; *p* = 0.015) and age over 60 years (HR: 2.231; CI: 1.245–4.001; *p* = 0.007) as independent predictors for OS. In conclusion, the addition of oxaliplatin to fluorouracil during nRCT significantly improved pCR-rate without having an impact on survival.

## 1. Introduction

Colorectal carcinomas (CRC) are among the three most common cancer types in Western industrialized countries [1,2]. Rectal cancer represents approximately 30 percent of all CRC, with men being affected more frequently than women. The median age at diagnosis is 69 years in men and 73 years in women, although an increase in incidence has been shown in patients younger than 50 years [3,4].

Neoadjuvant radiochemotherapy (nRCT) followed by total mesorectal excision (TME) is now the standard of care for locally advanced rectal cancer (LARC) that is located in the middle or lower rectum [5]. The well-established advantages of nRCT include a lower local recurrence rate as well as a higher sphincter preservation rate, especially for carcinoma in the lower rectum. Currently, as for simultaneous and adjuvant chemotherapy, the standard treatment for LARC is infusional fluorouracil (5-FU) or oral capecitabine, which is a prodrug of fluorouracil. Systemic treatment is usually completed with optional adjuvant systemic chemotherapy. A further goal besides preoperative tumor shrinkage with improved resectability is the early prevention of tumor spread [6], possibly achieved by intensification of systemic chemotherapy. The additional benefit of oxaliplatin in this setting has been debated for many years, and numerous randomized controlled trials have produced inconsistent results [7,8,9,10,11,12,13,14]. Further progress has been obtained with the adoption of total neoadjuvant therapy (TNT), which usually involves nRCT followed by consolidation chemotherapy prior to surgery [15,16,17,18,19,20,21,22,23]. Alternatively, induction chemotherapy can be performed prior to nRCT. TNT largely showed improvements in disease-free survival and overall survival. Furthermore, compared to classical nRCT, TNT can possibly enhance clinical and pathological complete response rates (c/pCR) to explore organ preservation. Nowadays, TNT is also an organ-preserving option, and patients can be closely monitored as “watch-and-wait” patients when complete clinical remission is achieved [24].

In the herein presented longitudinal study, “real world data” over a consecutive period of 15 years on the patterns of nRCT in LARC were collected with the primary question: Is the additional administration of oxaliplatin during nRCT beneficial in terms of pathologic complete remission (pCR), disease-free survival (DFS), and overall survival (OS)?

## 2. Materials and Methods

### 2.1. Patients and Eligibility Criteria

**Figure 1:**
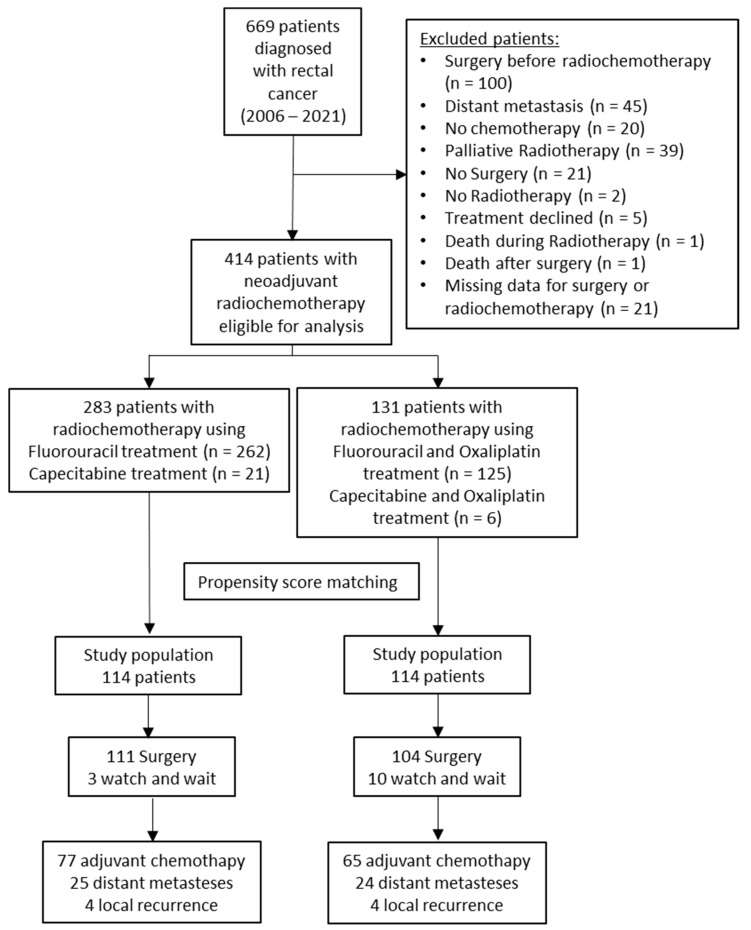
CONSORT flow diagram of the study population.

Between January 2006 and November 2021, a total of 669 patients with rectal cancer were treated at the Department of Radiation Oncology of the Coburg Cancer Centre and analyzed in this retrospective study. The CONSORT-diagram is shown in Figure 1. Exclusion criteria were (1) primary surgery, (2) evidence of distant metastases, (3) palliative radiotherapy, (4) no concurrent chemotherapy, (5) treatment refusal, (6) death during radiotherapy or surgery, and (7) insufficient data for surgery or RCT. A total of 414 patients with nRCT were identified and included in the study; 262 patients were treated by nRCT using concurrent chemotherapy with fluorouracil, 21 patients were treated using capecitabine, 125 patients were treated using a combination of fluorouracil and oxaliplatin, and 6 patients were treated with a combination of capecitabine and oxaliplatin. To reduce potential confounding effects between the fluorouracil/oxaliplatin treated group and the sole treatment with fluorouracil or capecitabine, propensity score matching analyses (PSM) were performed. In the matched cohort, 114 patients were included in each study population.

### 2.2. Study Procedure

Patients’ data were collected from clinical chart review, physician records, and patient correspondence. Parameters related to the treatment mainly included type and dose of chemotherapy, dose of radiotherapy, type of surgery, and type of adjuvant therapy. The last date of follow-up was 5 May 2022. The study, including further molecular analyses (which will be reported elsewhere), was approved by the Ethics Committee of the Friedrich-Alexander-University of Erlangen-Nürnberg on 15 August 2022 (Application No.: 22-166-B).

### 2.3. Outcome Measurement

The primary endpoint was complete clinical/pathologic response (c/pCR), defined as ypT0 N0 or uneventful watch-and-wait strategy. Secondary endpoint, overall survival (OS), was defined as time from histological diagnosis to death of any cause. Additional secondary endpoints included disease-free survival (DFS), which was defined as the time from histological diagnosis to one of the following events: non-radical surgery of the primary tumor (R2 resection), metastatic disease or locoregional recurrence, or death from any cause, whichever occurred first.

### 2.4. Statistical Analysis

Statistical analyses were performed using SPSS Statistics software package (IBM Corp. (USA), version 28.0.1.0). Categorical variables were distributed in terms of frequency and compared using the chi-squared test. Non-normally distributed continuous variables were compared using the Mann-Whitney U test. OS and DFS were assessed using de Kaplan-Meier method with log-rank test measuring the difference between the groups. The patients who were lost to follow-up were calculated according to survival at the last follow-up. Prognostic factors were assessed using a Cox-regression. The covariates that entered the analysis included gender, age, T- category, N-category, tumor location, Dworak regression grade, ECOG performance status, and the type of performed surgery. The propensity score matching analyses (PSM) were performed using the PSM module in the SPSS software (IBM Corp., version 28.0.1.0). The matching variables included age, clinical T-, N-category, and type of surgery. The adopted caliper of width was 0.02, and *p* < 0.05 was considered statistically significant.

## 3. Results

### 3.1. Patient Characteristics

There were 131 cases in the fluorouracil/oxaliplatin group and 283 in the fluorouracil group. Before PSM, the baseline characteristics of the two groups were different (Table 1). The median age of patients in the fluorouracil/oxaliplatin group was 59 years, and among patients in the fluorouracil group, it was 70 years (*p* < 0.001). A total of 228 patients (114 in each group) were successfully matched after PSM. Patient characteristics are shown in Table 1. There was no significant difference with respect to patient characteristics between the two matched groups.

### 3.2. Treatment

Radiotherapy was given as intensity-modulated treatment using 6-MV-photons in standard fractions of 1.8–2 Gy with a total dose of 50.4–56 Gy. Out of the 114 patients, a total of 104 (91%) patients were treated by nRCT using fluorouracil treatment, and capecitabine was used on 10 (9%) patients. A combination of fluorouracil and oxaliplatin was applied in 110 (97%) patients, and a combination of capecitabine and oxaliplatin was applied in 4 (4%) patients.

### 3.3. Surgical Outcome and Survival

Overall, 395 patients underwent surgery, with a low anterior resection being performed in the majority of 290 patients, and an abdominoperineal resection was performed in 105 patients. Before and after PSM, there was no statistically significant difference with respect to type of surgery (*p* = 0.206 and *p* = 0.896, respectively). The remaining 19 patients underwent a watch-and-wait regimen. The pathological results are shown in Table 1. After PSM, a statistically significant difference between the two patient groups was noted for the T-category only (*p* = 0.018). Combining the pathological complete response (pCR) cases with the patients without events in the watch-and-wait group, there were 18 c/pCR-patients in the fluorouracil-group and 31 patients in the fluorouracil/oxaliplatin group (*p* = 0.033 determined by Pearson’s chi-squared test).

**Figure 2:**
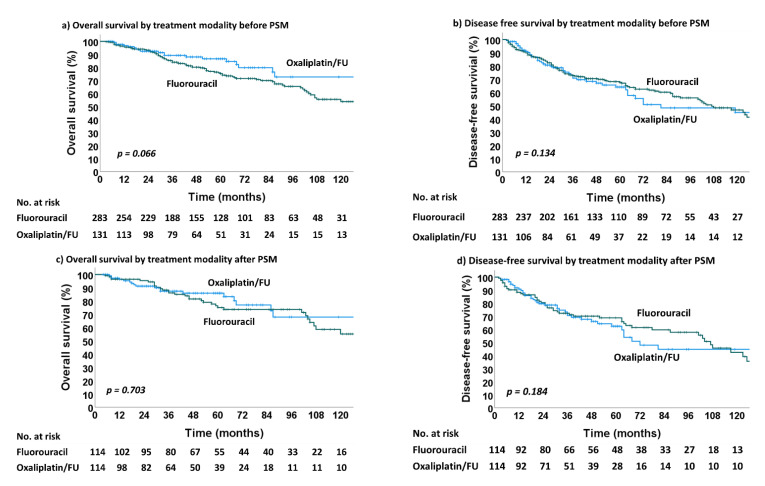
Overall survival and disease-free survival of the study population: (**a**) overall survival by treatment modality before PSM; (**b**) disease-free survival by treatment modality before PSM; (**c**) overall survival by treatment modality after PSM; (**d**) disease-free survival by treatment modality after PSM.

The median follow-up time after PSM was 59.5 weeks in the fluorouracil-group and 43 weeks in the fluorouracil/oxaliplatin group (*p* = 0.003). The median disease-free survival time was also statistically significant, being 47 weeks in the fluorouracil group and 32 weeks in the fluorouracil/oxaliplatin group (*p* = 0.035). As shown in Figure 2, there was no statistically significant difference between these two groups for both OS and DFS neither before nor after propensity score matching (72.6% vs. 55.4%, *p* = 0.066 and 67.8% vs. 55.1%, *p* = 0.703, respectively, for OS, and 44.8% vs. 46.8%, *p* = 0.134, and 44.7% vs. 42.3%, *p* = 0.184, respectively, for DFS). After PSM, local recurrence was reported in 4 cases in each treatment group (*p* = 1), and distant metastatic events were reported in 25 (22%) and 24 (21%) patients of the fluorouracil group and the fluorouracil/oxaliplatin group, respectively (*p* = 0.872).

### 3.4. Analysis of Prognostic Factors

**Figure 3:**
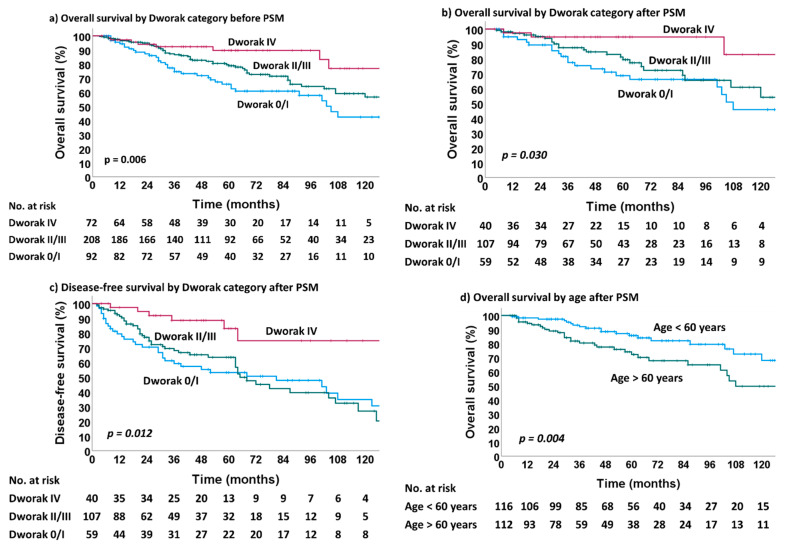
Overall survival and disease-free survival of the study population: (**a**) overall survival by Dworak category before PSM; (**b**) overall survival by Dworak category after PSM; (**c**) disease-free survival by Dworak category after PSM; (**d**) overall survival by age after PSM.

A subgroup analysis of survival outcomes indicated that tumor regression was a strong predictor for overall survival and disease-free survival. For further analysis, we used the three-tier tumor grouping established by Fokas et al. [25] (complete regression, Dworak IV; intermediate regression, Dworak II & III; and poor regression, Dworak 0 and I). As shown in Figure 3, the overall survival rates before and after PSM were significantly influenced by regression grading (*p* = 0.006 and *p* = 0.030). An impact on DFS was seen after PSM (*p* = 0.012). The patient group with an age below 60 years had a better overall survival (*p* = 0.004).

The results of univariate and multivariate analyses are given in Table 2. The factors with independent influence of overall survival were “age at diagnosis” and regression grading. Patients aged over 60 years had an HR of 2.255 (CI: 1.277–3.984) with a *p*-value of 0.005. Improved HR was associated with a Dworak grade of 2 or 3 and Dworak 4 (HR: 0.741; CI: 0.413–1.331 and HR: 0.460; CI: 0.251–0.841; *p*-value: 0.012, respectively).

### 3.5. Toxicity

As for the acute treatment-related toxicity of grade 3+, an increased rate of polyneuropathy was observed in pts of the oxaliplatin treatment group (12 vs. 0 pts). Mucositis of grade 3+ was seen primarily in the fluorouracil group (9 vs. 1 pts), and the rate of diarrhea requiring treatment was again increased in the oxaliplatin group (13 vs. 7 pts). One patient died during nRCT, which was due to acute myocardial infarction. Due to the fact that this is a retrospective analysis and only an evaluation of the documented patient data was possible, we have dispensed with a further statistical evaluation.

This section may be divided by subheadings. It should provide a concise and precise description of the experimental results, their interpretation, as well as the experimental conclusions that can be drawn.

## 4. Discussion

In the herein presented longitudinal study, “real world data” on the possible benefit of adding oxaliplatin to fluorouracil during nRCT in rectal cancer are presented. Both an enhanced pCR-rate (14% vs. 20%) as well as a higher proportion of watch-and-wait patients with sustained clinical complete remission were identified following the application of additional oxaliplatin (3% vs. 9%). When combining the pCR-patients and the watch-and-wait patients without recurrence, a significant advantage for oxaliplatin-based treatment was achieved (16% vs. 27%, *p* = 0.033). Albeit, neither an additional benefit on overall survival, and disease-free survival, nor a reduction in local recurrence rates or distant metastases could be demonstrated. This holds true also after propensity score matching analyses comparing two patient groups of 114 patients each with equally distributed characteristics and prognostic factors.

In a recent meta-analysis by Des Guetz et al. [26], it was shown that the addition of oxaliplatin had a significant benefit in terms of disease-free survival, pathologic complete response, and the reduction of distant metastasis. However, a benefit in overall survival could not be demonstrated. Of particular note, the CAO/ARO/AIO-04 trial [7] was the only large study to show a significant benefit in disease-free survival. On the other hand, the American NSABP R04 [8,9], the Italian STAR- 1 [10], the French ACCORD-12 [11,12,13], and the European PETACC-6 [14] trials failed to show any benefit. A significant improvement in DFS was surprisingly achieved by pooling the results from these studies [26]. Furthermore, the meta-analysis found a highly significant increase in toxicity in the oxaliplatin group, especially gastrointestinal toxicity. Table 3 provides an overview of all important studies that have investigated the impact of neoadjuvant therapy on the treatment of rectal cancer.

The most recent studies using a combination of fluorouracil and oxaliplatin are being conducted according to the scheme of total neoadjuvant therapy (TNT). The goal of TNT was to achieve an improvement in DFS and possibly a survival benefit. This has been shown in some studies, such as the RAPIDO [15], PRODIGE [18], STELLAR [19], and the American NCT00335816 study [20,21]. For example, the RAPIDO study demonstrated that TNT significantly reduced the rate of distant metastases (20% vs. 26.8%; HR 0.69; *p* = 0.019). Furthermore, the rate of pathological complete remission was doubled (28% vs. 14%; OR 2.37; *p* < 0.001). However, the rate of local recurrence was higher in the experimental group using the 5 × 5 Gy scheme (8.3% vs. 6.0%; HR 1.42; *p* = 0.12). This may be attributed to the possibly less effective short-course irradiation treatment. The standard treatment group received a total dose of 50.4Gy or 50Gy in 25–28 fractions. The PRODIGE study using induction chemotherapy with FOLFIRINOX found an improvement in pCR-rate (27.5% vs. 11.7%; *p* < 0.001) and a prolongation of DFS (3-year DFS: 75.7% vs. 68.5%; HR = 0.69; *p* = 0.034). A survival benefit could not be shown (3-year OS: 91% vs. 88%; HR = 0.65; *p* = 0.077). In accordance with the RAPIDO study, a significant reduction in distant metastases (17% vs. 25%) was seen. However, in contrast to RAPIDO, the rate of local recurrence was also lower in this study (4% vs. 6%, HR: 0.78; *p* = 0.56). This could also be due to a standard radiation treatment with 50Gy in 25 fractions administered in both arms. However, the question, which RT-regime is to be preferred (5 × 5 Gy or 28 × 1.8 Gy) is currently being investigated in the ongoing ACO/ARO/AIO 18.1 study. The STELLAR study found a significant benefit for treatment with TNT using capecitabine and oxaliplatin together with a short course radiotherapy. Here, an increased pCR + cCR rate was shown (21.8% vs. 12.3%; *p* = 0.002). The 3-year DFS was almost identical (64.5% vs. 62.3%, HR = 0.88; NS), and a survival advantage was seen for the 3-year OS (86.5% vs. 75.1%; HR = 0.67; *p* = 0.036), although this could not be further specified in any subgroup analysis. The rate of distant metastases was almost the same in both groups (22.8% vs. 24.7%), and the rate of local recurrence was slightly higher in the control group (8.4% vs. 11.0%; *p* = 0.461). However, compared to the fluorouracil group in other studies, such as PRODIGE and RAPIDO or the study we conducted, a local recurrence rate of 11% at a median follow-up of 35 months is considered high (for comparison 6%, 6%, and 4%, respectively, in each control arm).

On the other hand, the Polish-II study [22,23] failed to achieve both an improvement in DFS and OS. Here, however, oxaliplatin was also used in both arms. All other studies used only fluorouracil or capecitabine in the control arm. Recent studies like the CAO/ARO/AIO-12 trial [27,28] or the OPRA trial [24] have investigated different therapy sequences of TNT. RCT followed by consolidation chemotherapy seems to be the preferred TNT sequence when organ preservation is the primary concern, without compromising OS, DFS, toxicity, or quality of life.

In summary, recent TNT studies have shown that improvement in DFS and OS is possible due to a better response rate to treatment and thus a possible pathological complete remission. We were also able to demonstrate this. Patients who achieved ypT0 N0 and a tumor regression grade of Dworak/Fokas 4 had significantly higher survival rates than all other patients. A very poor response to RCT (Dworak/Fokas 0–1) was associated with an increased rate of distant metastases and shorter overall survival. This was also shown by the CAO/ARO/AIO-04 trial [25,29]. In addition, clinical and pathologic complete remission seems a surrogate marker for improved survival rates, better quality of life, and reduced healthcare costs. A nonoperative management of rectal cancer after RCT was shown to be cost saving compared with immediate surgery. This holds true even with a 25% likelihood for local regrowth with subsequent salvage surgery [30]. Another very interesting method of nonoperative treatment is the combination of external-beam radiation therapy followed by high-dose-rate brachytherapy. This has been shown to be feasible and well tolerated, especially in elderly and frail patients [31]. Nowadays, even a peritumoral abscess or fistula is no longer an absolute contraindication for RCT. These patients can be safely offered nRCT without an increase in toxicity and with the same pCR-rate, OS, and DFS [32].

We are aware that this study has some limitations. First, this was a retrospective analysis of 414 patients before PSM and 228 patients after PSM. Second, due to the lack of data on some patients (e.g., ECOG performance status, tumor grading), we were unable to perform further analysis on some parameters. This is mostly owing to the very long time between primary therapy and the time of this analysis. Third, the applied chemotherapy-regimens differed between the patients treated with 5-FU. The first-generation treatment was a continuous infusion of 5-FU with 1000 mg/m^2^ on days 1–5 and 29–33 during RT and four cycles of postoperative bolus 5-FU with 500 mg/^2^ according to Sauer et al. [5]. The other option was a modified O’Connel scheme with infusional 5-FU 225 mg/m^2^ for five weeks during RT. The other possibility was the application of total neoadjuvant therapy using 5-FU and oxaliplatin during nRCT and FOLFOX thereafter according to the CAO/ARO/AIO-12 study. Fourthly, the documentation of toxicity is mostly insufficient and limited to grades 3, 4, and 5. Thus, the events mentioned in the study were not discussed and compared with international studies. Finally, the follow-up time between the patients receiving 5-FUOX/CAPOX regimen and the 5-FU-based treatment differed significantly, with 43 months (IQR, 23–65) and 59.5 months (IQR, 33–103), respectively, which may be regarded as a possible confounder, even after PSM-analysis.

## 5. Conclusions

In summary, the results of our study indicate that the addition of oxaliplatin to a fluorouracil-based nRCT did improve pCR- and cCR-rates significantly, albeit not being able to demonstrate a clear survival benefit after propensity score matching.

## Figures and Tables

**Table 1:** Patient characteristics before and after PSM.

Characteristics	Before PSM (n = 414)			After PSM (n = 228)		
	5-FU & Cap. (n = 283)	5-FU/Oxa. (n = 131)	*p*-Value	5-FU & Cap. (n = 114)	5-FU/Oxa. (n = 114)	*p*-Value
**Sex**						
Female	86 (30%)	42 (32%)	0.732 ^1^	31 (27%)	34 (30%)	0.660 ^1^
Male	197 (70%)	89 (68%)		83 (73%)	80 (70%)	
**Age (years)**						
Median (IQR)	70 (61–75)	59 (53–66)	< 0.001 ^2^	59 (54–68)	61 (56–67)	0.568 ^2^
Mean (SD)	67 (10.0)	59 (9.0)		60 (10.0)	61 (8.7)	
**Clinical T category**						
cT2	17 (6%)	8 (6%)	0.155 ^1^	5 (5%)	8 (7%)	0.240 ^1^
cT3	223 (79%)	93 (71%)		91 (80%)	80 (70%)	
cT4	43 (15%)	30 (23%)		18 (16%)	26 (23%)	
**Clinical N category**						
cN0	77 (27%)	28 (21%)	0.342 ^1^	23 (20%)	25 (22%)	0.531 ^1^
cN1	60 (21%)	24 (18%)		28 (25%)	21 (18%)	
cN2	140 (50%)	77 (59%)		63 (55%)	67 (59%)	
cN3	1 (<1%)	1 (<1%)		0	1 (<1%)	
Missing data	5 (2%)	1 (<1%)		0	0	
**Location from anal verge**						
0–6 cm	148 (52%)	74 (57%)	0.443 ^1^	65 (57%)	66 (58%)	0.914 ^1^
7–12 cm	125 (44%)	52 (40%)		43 (38%)	44 (39%)	
>12 cm	3 (1%)	4 (3%)		2 (2%)	3 (3%)	
Missing data	7 (3%)	1 (<1%)		4 (4%)	1 (<1%)	
**Tumor differentiation**						
Well differentiated (G1)	12 (4%)	2 (2%)	0.443 ^1^	2 (2%)	2 (2%)	0.748 ^1^
Moderately differentiated (G2)	215 (76%)	97 (74%)		84 (74%)	82 (72%)	
Poorly differentiated (G3)	19 (7%)	6 (5%)		8 (7%)	5 (5%)	
Undifferentiated (G4)	1 (<1%)	0		0	0	
Missing data	36 (13%)	26 (20%)		20 (18%)	25 (22%)	
**Concurrent chemotherapy during preoperative radiotherapy**						
Fluorouracil	262 (93%)			104 (91%)		
Fluorouracil and Oxaliplatin		125 (95%)			110 (97%)	
Capecitabine	21 (7%)			10 (9%)		
Capecitabine and Oxaliplatin		6 (5%)			4 (4%)	
**Preoperative radiotherapy**						
Dose in Gy (mean with SD)	55 (1.6)	55 (4.6)	0.053 ^2^	55 (1.7)	55 (4.9)	0.027 ^2^
Duration in days (mean with SD)	38 (4.2)	39 (6.4)	0.902 ^2^	38 (3.6)	38 (6.7)	0.966 ^2^
**Chemotherapy dose**						
Fluorouracil in g (median with IQR)	10,000 (7875–10,000)	7000 (7000–14,200)	<0.001 ^2^	10,000 (7875–10,000)	7000 (7000–14,200)	<0.001 ^2^
Oxaliplatin in mg (median with IQR)		200 (200–385)	<0.001 ^2^		200 (200–377.5)	<0.001 ^2^
**Type of surgery**						
Abdominoperineal resection	71 (25%)	34 (26%)	0.206 ^1^	34 (30%)	31 (27%)	0.896 ^1^
Low anterior resection	203 (71%)	87 (66%)		77 (68%)	73 (64%)	
Watch and wait	9 (3%)	10 (8%)		3 (3%)	10 (9%)	
**Time between chemoradiotherapy and surgery in days (median and IQR)**	45 (41–54)	50 (42–68)	< 0.001^2^	46 (42–55)	50 (43–68)	0.005 ^2^
**Pathological T stage**	**n = 274**	**n = 121**		**n =111**	**n = 104**	
ypT0	45 (16%)	25 (21%)	0.080 ^1^	16 (14%)	22 (21%)	0.018 ^1^
ypT1	16 (6%)	5 (4%)		6 (5%)	3 (3%)	
ypT2	74 (27%)	30 (25%)		28 (25%)	24 (23%)	
ypT3	125 (46%)	46 (38%)		59 (53%)	42 (40%)	
ypT4	13 (5%)	14 (12%)		2 (2%)	12 (12%)	
Missing data	1 (<1%)	1 (<1%)		0	1 (<1%)	
**Pathological N stage**						
ypN0	209 (76%)	82 (68%)	0.341 ^1^	85 (77%)	71 (68%)	0.504 ^1^
ypN1	44 (16%)	23 (19%)		16 (14%)	20 (19%)	
ypN2	21 (8%)	13 (11%)		10 (9%)	11 (11%)	
Missing data	0	3 (2%)		0	2 (2%)	
**Completeness of local tumor resection**						
R0	260 (95%)	115 (95%)	0.740 ^1^	106 (96%)	99 (95%)	0.264 ^1^
R1	5 (2%)	1 (<1%)		4 (4%)	1 (<1%)	
R2	3 (1%)	1 (<1%)		0	1 (<1%)	
Missing data	6 (2%)	4 (4%)		1 (<1%)	3 (3%)	
**Dworak grade**						
0	7 (3%)	3 (3%)	0.794 ^1^	5 (5%)	3 (3%)	0.566 ^1^
1	59 (22%)	23 (19%)		30 (27%)	21 (20%)	
2	94 (34%)	47 (39%)		40 (36%)	40 (39%)	
3	48 (18%)	19 (16%)		13 (12%)	14 (14%)	
4	46 (17%)	26 (22%)		17 (15%)	23 (22%)	
Missing data	20 (8%)	3 (3%)		6 (5%)	3 (3%)	
**Pathological complete response**						
ypT0N0	42 (15%)	24 (20%)	0.260 ^1^	15 (14%)	21 (20%)	0.179 ^1^
**Adjuvant chemotherapy**						
Yes	179 (63%)	75 (57%)	0.151 ^1^	77 (68%)	65 (57%)	0.042 ^1^
No	96 (34%)	55 (42%)		32 (28%)	48 (42%)	
Missing data	8 (3%)	1 (<1%)		5 (4%)	1 (<1%)	
**Type of adjuvant chemotherapy**						
Fluorouracil	135 (71%)	41 (51%)	< 0.001 ^1^	54 (66%)	38 (54%)	0.016 ^1^
Fluorouracil and Oxaliplatin	14 (7%)	25 (31%)		9 (11%)	20 (28%)	
Palliative chemotherapy	7 (4%)	2 (3%)		1 (1%)	2 (3%)	
Others	16 (8%)	6 (7%)		11 (13%)	4 (6%)	
Missing data	17 (9%)	7 (9%)		7 (9%)	7 (10%)	
**Distant metastases**						
Yes	53 (19%)	29 (22%)	0.418 ^1^	25 (22%)	24 (21%)	0.872 ^1^
No	230 (81%)	102 (78%)		89 (78%)	90 (79%)	
**Local recurrence**						
Yes	13 (5%)	5 (4%)	0.718 ^1^	4 (4%)	4 (4%)	1 ^1^
No	270 (95%)	126 (96%)		110 (96%)	110 (96%)	
**Median follow up time in months with IQR**	53 (29–91)	46 (24–69)	0.054^2^	59,5 (33–103)	43 (23–65)	0.003 ^2^
**Median DFS time in months with IQR**	**44 (20–86)**	**34 (18–61)**	**0.044 ^2^**	**47 (20–94)**	**32 (15–60)**	**0.035 ^2^**

*p*-values were determined by ^1^ Chi-squared Test or ^2^ Mann Whitney U Test. 5-FU & Cap.: Treatment with Fluorouracil or Capecitabine. 5-FU/Oxa.: Treatment with Fluorouracil/Capecitabine and Oxaliplatin

**Table 2:** Prognostic factors: Univariate and multivariate analysis.

Characteristic		Univariate Analysis			Multivariate Analysis		
	Cases	*p*-Value	HR	95%CI	*p*-Value	HR	95%CI
**Sex**							
Male	163	Reference	1	Reference			
Female	65	0.550	0.825	0.439–1.549			
**T stage**							
T2–3	184	Reference	1	Reference			
T4	44	0.878	0.943	0.442–2.009			
**Tumor location**							
0–6 cm	131	Reference	1	Reference			
7–13 cm	87	0.440	0.807	0.468–1.391			
**Age**							
<60 years	116	Reference	1	Reference	Reference	1	Reference
>60 years	112	0.005	2.255	1.277–3.984	0.007	2.231	1.245–4.001
**N stage**							
N0	48	Reference	1	Reference			
N+	180	0.648	1.165	0.606–2.238			
**Dworak grade**							
Dworak 0 & 1	59	Reference	1	Reference	Reference	1	Reference
Dworak 2 & 3	107	0.316	0.741	0.413–1.331	0.165	0.786	0.559–1.104
Dworak 4	40	0.012	0.460	0.251–0.841	0.015	0.659	0.471–0.921
**ECOG performance status**							
ECOG 0	77	Reference	1	Reference			
ECOG > 0	52	0.884	1.066	0.454–2.501			
**Type of Surgery**							
Abdominoperineal resection	65	Reference	1	Reference			
Low anterior resection	150	0.390	1.287	0.724–2.289			

**Table 3:** Clinical trials that investigate the Role of Oxaliplatin-Based RCT and TNT (Cf. [14,26,33]).

Trial No. of Patients	Preoperative Treatment	Postoperative Treatment	Results
CAO/ARO/AIO-04 1236	Control: RT-1 + 5FU 1000 mg/m^2^ days 1–5 and 29–33 Exp: RT-1 + 5FU 250 mg/m^2^ days 1–14 and 22–35 + Ox 50 mg/m^2^ days 1, 8, 22, 29	Control: 5FU Exp: FOLFOX	pCR 13% vs. 17%; *p* = 0.038 3-year DFS: 71.2% vs. 75.9% HR = 0.79; *p* = 0.03 3-year OS: 88.0% vs. 88.7% HR = 0.96; NS
NSABP R04 1608	RT (45Gy + 5.4 or 10.8 boost) + Control: 5FU 225 mg/m^2^/d or CaP 825 mg/m^2^ twice daily Exp: 5FU 225 mg/m^2^/d or CAPOX 825 mg/m^2^ twice daily + Ox 50 mg/m^2^ weekly for 5 w	Open	pCR 17.8% vs. 19.5%; *p* = 0.42 5-year DFS: 64.2% vs. 69.2% HR = 0.91; *p* = 0.34 5-year OS: 79% vs. 81.3% HR = 0.89; *p* = 0.38
STAR-1 747	CL: RT-1 + 5FU 225 mg/m^2^/d Exp: RT-1 + 5FU 225 mg/m^2^/d + Ox 60 mg/m^2^ weekly for 6 w	Any 5FU based	pCR 16% vs. 16%; *p* = 0.9
ACCORD-12 598	Control: RT (45Gy; 1.8) + CaP 800 mg/m^2^ twice daily Exp: RT (50Gy; 2.0) + CaP 800 mg/m^2^ twice daily + Ox 50 mg/m^2^/w	Open	pCR 13.9% vs. 19.2%; *p* = 0.09 3-year DFS: 67.9% vs. 72.7%. HR = 0.88; *p* = 0.39 3-year OS: 87.6% vs. 88.3%. HR = 0.94; NS
PETTAC-6 1094	RT 45Gy (1.8) or 50.4 (1.8) + Control: CaP 825 mg/m^2^ 2xd Exp: CaP 825 mg/m^2^ twice daily + Ox 50 mg/m^2^/d Weekly for 5 w	Control: Cape Exp: CAPOX	pCR 11.5% vs. 13.5%; *p* = 0.31 5-year DFS: 71.3% vs. 70.5%; HR = 1.03; *p* = 0.78 5-year OS: 83.1% vs. 80.1%; HR = 1.19; *p* = 0.2
RAPIDO 912	CL: RT 50.4 Gy (1.8) or 50 Gy (2.0) + CaP 825 mg/m^2^ 2xd Exp: SCPRT + 9 cycles FOLFOX4 (Ox 85 mg/m^2^) or 6 cycles CAPOX (Ox 130 mg/m^2^)	Optional (12 cycles FOLFOX4 or 8 cycles CAPOX)	pCR: 28% vs. 14%; *p* < 0.001 3-year Disease-related treatment failure: 24% vs. 30%; *p* = 0.019 3-year OS: 89.1% vs. 88.8%; HR = 0.92; *p* = 0.59
PRODIGE 461	RT 50 Gy (2.0) + Control: CaP 800 mg/m^2^ Exp: 6 cycles FOLFIRINOX (Ox 85mg/m^2^, Irinotecan 180 mg/m^2^, Leucovorin 400 mg/m^2^)	mFOLFOX or capecitabine	pCR: 27.5% vs. 11.7%; *p* < 0.001 3-year DFS: 75.7% vs. 68.5%; HR = 0.69; *p* = 0.034 3-year OS: 91% vs. 88%; HR = 0.65; *p* = 0.077
STELLAR 599	Control: RT 50 Gy (2.0) + CaP 825 mg/m^2^ twice daily Exp: SCPRT + 4 × CAPOX (Ox 130 mg/m^2^ + CaP 1000 mg/m^2^ twice daily)	CAPOX 2 cycles in TNT 6 cycles in Control	pCR + cCR rate: 21.8% vs. 12.3%; *p* = 0.002 3-year DFS: 64.5% vs. 62.3%, HR = 0.88; NS 3-year OS: 86.5% vs. 75.1%; HR = 0.67; *p* = 0.036
NCT00335816 259	RT 45 Gy (1.8) + boost 5.4 Gy+ Control: 5FU 225 mg/m^2^/d Exp: 5FU 225 mg/m^2^/d + FOLFOX6 (Ox 85 mg/m^2^ weekly for 2, 4 or 6 w	Open	pCR: 18%, 25%, 30%, and 38% in Arms 1, 2, 3, or 4; *p* = 0.0036 5-year DFS: 50%, 81%, 86%, and 76% in Arms 1, 2, 3, and 4; *p* = 0.004 5-year OS: 79%, 92%, 88%, and 84% in Arms 1, 2, 3, and 4; NS
POLISH-II 515	CL: RT 50.4 Gy (1.8) + 5FU 325 mg/m^2^/d for 2w, Ox 50 mg/m^2^/d for 5 w Exp: SCPRT + 3 cyc FOLFOX4	Open	pCR: 16% vs. 12%; *p* = 0.17 8-year DFS: 43% vs. 41%; HR = 0.95; *p* = 0.65 8-year OS: 49% vs. 49%; NS

## Data Availability

Datasets will not be publicly provided due to privacy of the patients.

## Abbreviations

5FU, fluorouracil; 5FUOX, fluorouracil and oxaliplatin; Cape, capecitabine; CAPOX, capecitabine and oxaliplatin; CL, Control; DFS, disease-free survival; FOLFOX; infusional fluorouracil, leucovorin, and oxaliplatin; HR, hazard ratio; NS, not significant; OS, overall survival; Ox, oxaliplatin; pCR, pathologic complete remission; cCR, clinical complete response; RT, radiotherapy: RT-1: = 50.4 Gy (1.8); SCPRT, short-course preoperative radiotherapy (5 × 5 Gy).
